# Sarcopenia is associated with short‐ and long‐term mortality in patients with acute‐on‐chronic liver failure

**DOI:** 10.1002/jcsm.13501

**Published:** 2024-07-05

**Authors:** Fan Zeng, Wei Jiang, Xiujun Chang, Fuxun Yang, Xiaoxiu Luo, Rongan Liu, Yu Lei, Jiajia Li, Chun Pan, Xiaobo Huang, Huaiqiang Sun, Yunping Lan

**Affiliations:** ^1^ Department of Intensive Care Unit Sichuan Academy of Medical Sciences and Sichuan Provincial People's Hospital Chengdu China; ^2^ Clinical Medicine School of Chengdu University of Traditional Chinese Medicine Chengdu China; ^3^ Huaxi MR Research Center (HMRRC), Department of Radiology West China Hospital of Sichuan University Chengdu China

**Keywords:** acute‐on‐chronic liver failure, mortality, retrospective studies, sarcopenia

## Abstract

**Background:**

While sarcopenia is recognized as a predictor of mortality in cirrhosis, its influence on acute‐on‐chronic liver failure (ACLF) remains uncertain. Despite multiple studies examining the impact of sarcopenia on short‐term mortality in patients with ACLF, the sample size of these studies was limited, and their outcomes were inconsistent. Therefore, this study aimed to explore the impact of sarcopenia on both short‐ and long‐term mortality in patients with ACLF.

**Methods:**

This retrospective cohort study included 414 patients with ACLF that were treated between January 2016 and September 2022. Sarcopenia was diagnosed based on the measurement of the skeletal muscle index at the third lumbar vertebra (L3‐SMI). Subsequently, the patients were divided into sarcopenia and non‐sarcopenia groups. We analysed the basic clinical data of the two groups. Multivariate Cox proportional analysis was used to analyse short‐term (28 days) and long‐term (1 year and overall) mortality rates.

**Results:**

A total of 414 patients were included, with a mean age of 52.88 ± 13.41 years. Among them, 318 (76.8%) were male, and 239 (57.7%) had sarcopenia. A total of 280 (67.6%) patients died during the study period. Among them, 153 patients died within 28 days (37%) and 209 patients died within 1 year (50.5%). We found that the 28‐day, 1‐year and overall mortality rates in the sarcopenia group were significantly higher than those in the non‐sarcopenia group (37% vs. 22.3%, *P* < 0.01; 50.5% vs. 34.9%, *P* < 0.01; and 67.6% vs. 53.1%, *P* < 0.01, respectively). Multivariate Cox regression analysis revealed that sarcopenia was significantly associated with increased mortality. The hazard ratios for sarcopenia were 2.05 (95% confidence interval [CI] 1.41–3.00, *P* < 0.01) for 28‐day mortality, 1.81 (95% CI 1.29–2.54, *P* < 0.01) for 1‐year mortality and 1.82 (95% CI 1.30–2.55, *P* < 0.01) for overall mortality. In addition, muscle density and international normalized ratio were associated with short‐ and long‐term mortality.

**Conclusions:**

Sarcopenia is associated with both short‐ and long‐term mortality in patients with ACLF. Therefore, regular monitoring for sarcopenia is important for these patients.

## Introduction

Acute‐on‐chronic liver failure (ACLF) refers to a clinical syndrome characterized by the abrupt deterioration of liver function in individuals with an underlying chronic liver disease. This condition is characterized by the manifestation of liver failure and/or failure of other organs outside the liver (extrahepatic organ failure). ACLF is associated with a high risk of short‐term mortality, emphasizing the urgency for prompt diagnosis, appropriate management and close monitoring of these patients.^1^ Despite advancements in medical science, ACLF continues to pose a formidable mortality challenge.^2^ In China, the predominant cause of ACLF is hepatitis B virus‐associated ACLF (HBV‐ACLF), which carries a high pre‐transplant mortality rate ranging from 60% to 75%.^3^ Every year, an estimated 120 000 lives are lost owing to HBV‐ACLF in the Asia‐Pacific region.^4^ Unlike cirrhosis, the progression of ACLF is driven by a central role played by systemic inflammation. An intense inflammatory response triggers oxidative stress, inducing a sustained state of heightened metabolic activity within the body. This metabolic dysregulation results in insulin resistance, increased protein catabolism and a persistent negative nitrogen balance, all of which predispose individuals to the risk of muscle atrophy.^5^ Research has revealed that patients with ACLF exhibit elevated glycolysis, mitochondrial dysfunction, reduced ATP production, impaired muscle regeneration and a more pronounced acceleration of muscle breakdown, distinguishing these patients from those with decompensated cirrhosis.^6^

Sarcopenia, a term derived from the Greek ‘sarco’ (flesh) and ‘penia’ (deficiency), refers to the age‐related loss of skeletal muscle. Sarcopenia is defined as a critical reduction in muscle mass.^7^ A growing number of studies have revealed a correlation between sarcopenia and unfavourable clinical outcomes in patients with sepsis, whose pathogenesis is also driven by inflammatory responses.^8^ Despite numerous investigations into the influence of sarcopenia in patients with cirrhosis, acknowledging that the pathogenesis and metabolism of ACLF are different is important. The precise effect of muscle deficiency in patients with ACLF remains uncertain.

The assessments of ACLF conducted in previous studies are limited, and their conclusions are often contradictory.^9^, ^10^ Moreover, these studies primarily focused on short‐term mortality and had relatively small sample sizes.^11^ Therefore, we sought to explore the association between sarcopenia and short‐ and long‐term mortality in a substantial patient population with ACLF in this study.

## Methods

### Research participants

For this retrospective investigation, we enrolled individuals diagnosed with ACLF who were admitted to the Sichuan Provincial People's Hospital between January 2016 and September 2022 and met the diagnostic criteria outlined by the Asian Pacific Association for the Study of the Liver (APASL). All patients received a standardized medical care regimen during hospitalization, including energy supplementation, prophylactic measures against bacterial infections, treatment of associated complications and antiviral therapy. We conducted a retrospective analysis of the clinical data and survival status of all patients adhering to the specific inclusion criteria. ACLF was defined as an acute hepatic insult characterized by jaundice (serum bilirubin levels ≥ 5 mg/dL) and coagulopathy (international normalized ratio [INR] ≥ 1.5), occurring within 4 weeks and accompanied by clinical ascites and/or encephalopathy in individuals with previously diagnosed or undiagnosed chronic liver disease or cirrhosis. These two conditions have a notable association with high 28‐day mortality.^12^

Patients who were aged <18 years, were pregnant and had undergone liver transplantation during their current hospitalization, and those for whom imaging data could not be obtained, were excluded from the study. All patients received standard medical treatment, including energy supplementation, intravenous administration of albumin and plasma, artificial liver support and preventive measures against post‐admission complications. Artificial liver support primarily involved plasma exchange combined with bilirubin adsorption or a double plasma molecular adsorption system. The specific approach was determined by the attending clinical physicians. Ethical approval for this study was granted by the Ethics Committee of Sichuan Provincial People's Hospital. We extracted fundamental clinical information from the hospital's electronic medical records, including details such as age, sex, laboratory and virology test results and the presence of major complications. Patient outcomes were ascertained via telephone contact or outpatient follow‐up. The calculation formulas for the model for end‐stage liver disease (MELD) score and the platelet–albumin–bilirubin (PALBI) score are as follows: MELD score = 3.8 × ln(serum bilirubin [mg/dL]) + 11.2 × ln(INR) + 9.6 × ln(serum creatinine [mg/dL]) + 6.4^13^ and PALBI score = 2.02 × log10[bilirubin (μmol/L)] − 0.37 × {log10[bilirubin (μmol/L)]}^2^ − 0.04 × albumin − 3.48 × log10(platelets) + 1.01 × {log10(platelets)}^2^.^14^ MELD scores were determined in accordance with prior research methodologies.^15^, ^16^

### Assessment of the skeletal muscle index at the third lumbar vertebra (L3‐SMI)

Skeletal muscle mass was evaluated by assessing the skeletal muscle index (SMI) using computed tomography (CT) performed at the L3 vertebral level. The skeletal muscle area (SMA) at the L3 vertebral level, represented by square centimetres, reflects the cross‐sectional area of the human skeletal muscle as observed on CT imaging. This area includes major muscle groups, such as the psoas muscle, vertical spinal muscles, abdominal internal oblique muscle, transverse abdominis muscle, abdominal external oblique muscle and rectus abdominis.

**Figure 1:**
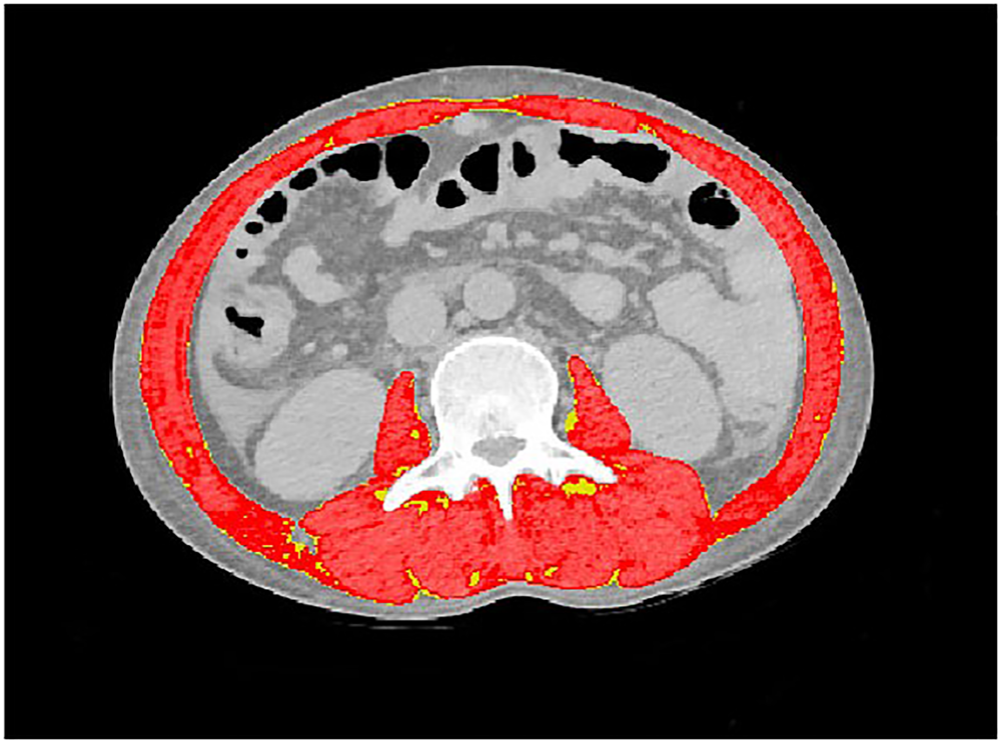
A representative computed tomography image at the L3 vertebral level for the quantification of skeletal muscle area (red) and intermuscular adipose tissue (yellow).

To ensure consistency and accuracy of muscle segmentation at the L3 vertebral level, this study employed an automatic segmentation approach based on deep learning. Training data preparation involved selecting the L3 vertebral level within the CT scans and manually outlining the muscle area at the L3 level using ITK‐SNAP. Thirty patients underwent manual delineation. For model training, the original CT slices and manually outlined L3 vertebral level mask were input into the nnUNet software package, utilizing default parameters throughout the training process. The developed segmentation model achieved an average DICE score of 0.937 for the training data. For model inference, the trained model was applied to generate muscle masks at the L3 level for all the remaining images. Subsequently, a radiologist with 10 years of experience in abdominal diagnosis conducted a thorough review and made the necessary manual corrections to the automatically generated masks. Distinguishing between muscle and fat was achieved by employing a threshold within the mask's range, with muscle defined as having a density between −29 and 150 Hounsfield units (HU) and fat between −30 and −190 HU, as previously reported in existing studies (*Figure* *1*).^17^ SMI was calculated using the following formula: SMI = SMA in square centimetres divided by the square of the body height in metres to account for the influence of body size.^18^

Sarcopenia was defined based on sex‐specific cut‐offs for the SMI. Myopaenia was diagnosed in female patients with an SMI < 39 cm^2^/m^2^ and in male patients with an SMI < 50 cm^2^/m^2^.^19^ Additionally, myosteatosis was identified by considering the lowest quartile (Q1) values of muscle density, which corresponded to values ≤18.9 HU for female patients and ≤28.1 HU for male patients, as per prior research.^20^

### Statistical analysis

Continuous variables were analysed using either Student's *t*‐test or the Mann–Whitney *U* test, as deemed appropriate for the data distribution. The results are presented as either the mean ± standard deviation or the median (25th and 75th percentiles). Categorical data were compared using the *χ* ^2^ test, and the outcomes were expressed as numbers (with corresponding percentages). The proportionality of hazards within the Cox model was assessed through residual assessments and graphical representations.

Furthermore, multivariate regression models were constructed, adjusting for confounding variables that were selected based on their clinical relevance among the risk factors with a significance level of *P* < 0.05 while considering the presence of multicollinearity.

Kaplan–Meier survival analyses and log‐rank tests were conducted to compare the short‐ and long‐term prognoses of patients with and without sarcopenia. Each patient was subject to a 1‐year follow‐up period after discharge. The threshold for statistical significance was set at *P* < 0.05. All statistical analyses were performed using SPSS (Version 22.0; IBM Corp., Armonk, NY, USA) and the R package (Version 3.4.3; R Foundation for Statistical Computing, Vienna, Austria).

## Results

**Figure 2:**
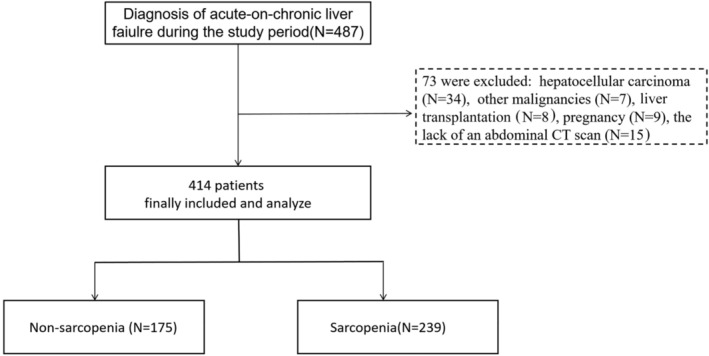
Flow diagram of the study population. CT, computed tomography.

Of the initial cohort of 487 patients recruited for this study, 73 were excluded from the analysis. Those excluded consisted of 34 patients with hepatocellular carcinoma, 7 with other extrahepatic malignancies, 8 slated for liver transplantation, 9 pregnant patients and 15 lacking abdominal CT scan data. Consequently, the final analysis included 414 patients diagnosed with ACLF (*Figure* *2*). The average age of this cohort was 52.88 ± 13.41 years and was composed of 318 males. Viral hepatitis (*n* = 275) was the predominant cause of ACLF, followed by alcohol‐related causes (*n* = 74).

Patients were then categorized into two groups: those with and without sarcopenia. Notably, the sarcopenia group had a higher proportion of male patients and older individuals than the non‐sarcopenia group. Laboratory findings revealed significant differences between the groups, with lower albumin levels and muscle density and higher Chronic Liver Failure‐Sequential Organ Failure Assessment (CLIF‐SOFA) scores in the sarcopenia group than in the non‐sarcopenia group. However, no significant differences were observed between the two groups in terms of complications such as acute respiratory distress syndrome (ARDS), hepatic encephalopathy and acute kidney injury (AKI) (*Table* *1*).

**Table 1:** General characteristics at baseline

Variables	Total (*N* = 414)	Non‐sarcopenia (*N* = 175)	Sarcopenia (*N* = 239)	*P*
Male (*N*, %)	318 (76.81)	122 (69.71)	196 (82.00)	0.00
Age (years)	52.88 ± 13.41	50.39 ± 12.90	54.68 ± 13.52	0.01
BMI (kg/m^2^)	23.73 ± 3.08	24.93 ± 3.23	22.85 ± 2.64	0.00
Laboratory findings				
WBC (*10^9^/L)	7.20 ± 4.52	6.86 ± 4.09	7.45 ± 4.79	0.19
Platelet (*10^9^/L)	98.07 ± 67.48	99.82 ± 67.63	96.79 ± 67.47	0.65
Total bilirubin (mg/dL)	13.74 ± 9.41	13.51 ± 8.85	13.90 ± 9.80	0.68
Albumin (g/L)	29.27 ± 5.58	30.04 ± 5.78	28.70 ± 5.37	0.02
Creatinine (mg/dL)	1.01 ± 1.17	0.89 ± 0.98	1.1 ± 1.3	0.09
AFP (ng/mL)	134.27 ± 370.13	113.87 ± 306.56	149.56 ± 411.53	0.38
Ammonia (μmol/L)	85.62 ± 63.91	83.54 ± 58.44	87.1 ± 67.6	0.54
INR	1.92 ± 0.67	1.88 ± 0.59	1.95 ± 0.72	0.3
L3‐SMI (cm^2^/m^2^)	45.43 ± 11.08	53.80 ± 10.45	39.30 ± 6.62	0.00
Myosteatosis	37.25 ± 8.58	38.50 ± 7.85	36.33 ± 8.99	0.01
Aetiology of ACLF (*N*, %)				
Virus (virus B, C)	275 (66.42)	131 (74.86)	144 (60.25)	0.02
Alcoholic hepatitis	13 (3.14)	6 (3.42)	7 (2.93)	0.78
Alcohol	74 (17.87)	26 (14.86)	48 (20.08)	0.19
Auto‐immune	11 (2.66)	3 (1.71)	8 (3.35)	0.37
Others	41 (9.90)	9 (5.14)	32 (13.39)	0.77
Complications (*N*, %)				
ARDS	117 (28.26)	43 (24.57)	74 (30.96)	0.15
Peritonitis	176 (42.51)	67 (38.29)	109 (45.61)	0.14
HE	87 (21.01)	30 (17.14)	57 (23.85)	0.10
Bleeding	109 (26.32)	39 (22.28)	70 (29.28)	0.11
AKI	91 (21.98)	30 (17.14)	61 (25.52)	0.04
Chronic disease (*N*, %)				
Cirrhosis	335 (80.92)	135 (77.14)	200 (83.68)	0.04
Cardiovascular disease	50 (12.08)	23 (13.14)	27 (11.30)	0.57
Diabetes	60 (14.49)	22 (12.57)	38 (15.90)	0.34
Predictive score				
MELD score	22.65 ± 5.32	22.41 ± 4.85	22.84 ± 5.64	0.41
CTP score	10.50 ± 1.65	10.33 ± 1.67	10.63 ± 1.62	0.07
PALBI score	11.77 ± 2.14	11.79 ± 2.14	11.75 ± 2.15	0.85
CLIF‐SOFA	5.80 ± 2.30	5.49 ± 2.36	6.03 ± 2.37	0.02
Mortality (*N*, %)				
28 days	153 (36.96)	39 (22.28)	114 (47.70)	0.00
1 year	209 (50.48)	61 (34.85)	148 (61.92)	0.00
Overall	280 (67.63)	93 (53.14)	187 (78.24)	0.00

*Note*: Mean ± standard deviation for the continuous variables; *N* (%) for the categorical variables. Abbreviations: ACLF, acute‐on‐chronic liver failure; AFP, alpha‐fetoprotein; AKI, acute kidney injury; ARDS, acute respiratory distress syndrome; BMI, body mass index; CLIF‐SOFA, Chronic Liver Failure‐Sequential Organ Failure Assessment; CTP score, Child–Turcotte–Pugh score; HE, hepatic encephalopathy; INR, international normalized ratio; L3‐SMI, skeletal muscle index at the third lumbar vertebra; MELD score, model for end‐stage liver disease score; PALBI score, platelet–albumin–bilirubin score; WBC, white blood cell.

**Figure 3:**
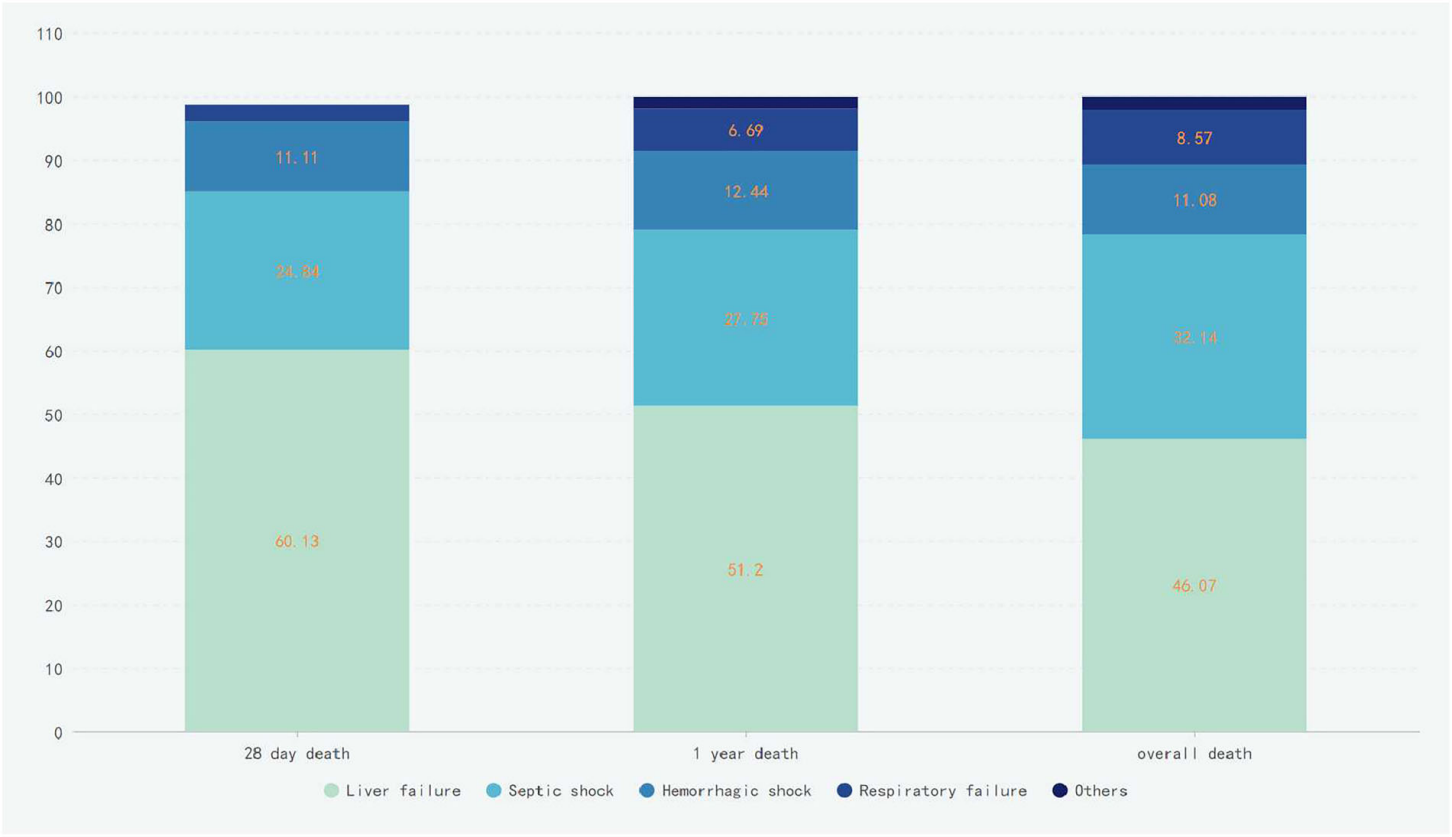
The causes of 28‐day, 1‐year and overall death in patients with acute‐on‐chronic liver failure.

In terms of mortality rates, the sarcopenia group had significantly higher 28‐day, 1‐year and overall mortality rates than the non‐sarcopenia group (*Table* *1*). During the follow‐up period until September 2023, 117 patients survived, while 280 patients succumbed to their condition. Among the deceased, 153 patients died within 28 days (37%), 209 died within 1 year (50.5%) and 17 were lost to follow‐up after the first year. The longest patient follow‐up exceeded 6 years, with a median follow‐up duration of 75.6 months among the surviving patients. Liver failure was the primary cause of death, followed by septic shock. Notably, the proportion of deaths attributable to septic shock gradually increased with extended survival time (*Figure* *3*). There was no difference in the proportion of deaths caused by sarcopenia between the sarcopenia and non‐sarcopenia groups (*Table* *2*), but there was a noticeable increase in deaths attributed to septic shock in the non‐sarcopenia group over time.

**Table 2:** Death causes of the patients in the 28‐day, 1‐year and overall mortality

	Total	Non‐sarcopenia	Sarcopenia	*P*
28‐day mortality (*N*, %)				
Liver failure	92 (60.13)	24 (61.54)	68 (59.64)	0.84
Septic shock	38 (24.84)	9 (23.07)	29 (25.43)	0.77
Haemorrhagic shock	17 (11.11)	5 (12.82)	12 (10.53)	0.69
Respiratory failure	4 (2.61)	1 (2.56)	3 (2.63)	0.98
Others	2 (1.31)	0 (0)	2 (0.17)	0.41
1‐year mortality (*N*, %)				
Liver failure	107 (51.20)	34 (55.74)	73 (45.32)	0.40
Septic shock	58 (24.84)	13 (21.13)	45 (30.41)	0.18
Haemorrhagic shock	26 (12.44)	10 (16.39)	16 (10.81)	0.27
Respiratory failure	14 (6.70)	2 (3.28)	12 (8.11)	0.21
Others	4 (1.91)	2 (3.28)	2 (1.35)	0.36
Overall mortality (*N*, %)				
Liver failure	129 (46.07)	41 (44.08)	88 (47.06)	0.64
Septic shock	90 (32.14)	24 (25.81)	66 (35.29)	0.11
Haemorrhagic shock	31 (11.07)	15 (16.13)	16 (8.56)	0.06
Respiratory failure	24 (8.57)	9 (9.68)	15 (9.09)	0.64
Others	6 (2.14)	4 (4.30)	2 (1.07)	0.08

For univariate and multivariate analyses of 28‐day, 1‐year and overall mortality, we conducted univariate Cox proportional hazards analyses. These analyses revealed that sarcopenia, age, albumin, creatinine, INR, muscle density, ARDS, hepatic encephalopathy, AKI, MELD score, Child–Turcotte–Pugh (CTP) score and CLIF‐SOFA score were significantly associated with mortality outcomes in patients with ACLF. Moreover, body mass index (BMI) was linked to increased 1‐year and overall mortality, whereas gastrointestinal bleeding was associated with increased 28‐day and overall mortality (*Table* *3*).

**Table 3:** Univariable and multivariable analyses of sarcopenia for the 28‐day, 1‐year and overall mortality

Variables	28‐day mortality				1‐year mortality				Overall mortality			
	Univariable		Multivariable		Univariable		Multivariable		Univariable		Multivariable	
	HR (95% CI)	*P*	HR (95% CI)	*P*	HR (95% CI)	*P*	HR (95% CI)	*P*	HR (95% CI)	*P*	HR (95% CI)	*P*
Age (years)	1.02 (1.00, 1.03)	<0.01	1.00 (0.99, 1.02)	0.41	1.02 (1.01, 1.03)	0.00	1.01 (1.00, 1.02)	0.28	1.02 (1.01, 1.03)	<0.01	1.01 (0.99, 1.02)	0.27
BMI	0.97 (0.92, 1.02)	0.20			0.96 (0.92, 1.00)	0.02	1.00 (0.99, 1.00)	0.50	0.95 (0.92, 0.99)	0.01	0.97 (0.92, 1.00)	0.25
WBC (*10^9^/L)	1.03 (1.00, 1.06)	0.58			1.02 (0.99, 1.05)	0.17			1.02 (1.00, 1.05)	0.08		
TB (mg/dL)	1.01 (0.99, 1.02)	0.44			1.00 (0.99, 1.02)	0.71			1.00 (1.00, 1.02)	0.22		
Albumin (g/L)	0.95 (0.92, 0.98)	<0.01	0.97 (0.93, 1.00)	0.05	0.96 (0.93, 0.98)	<0.01	0.98 (0.95, 1.01)	0.15	0.96 (0.94, 0.98)	<0.01	0.98 (0.95, 1.01)	0.14
Creatinine (mg/dL)	1.12 (1.01, 1.23)	0.02	1.02 (0.88, 1.19)	0.76	1.10 (1.00, 1.21)	0.04	1.01 (0.87, 1.17)	0.89	1.01 (1.01, 1.20)	0.20		
INR	1.70 (1.40, 2.06)	<0.01	1.63 (1.34, 1.91)	<0.01	1.65 (1.38, 2.00)	<0.01	1.62 (1.34, 1.97)	<0.01	1.65 (1.39, 1.95)	<0.01	1.64 (1.36, 1.99)	<0.01
Ammonia (μmol/L)	1.00 (1.00, 1.00)	0.03			1.00 (1.00, 1.00)	<0.01			1.00 (1.00, 1.00)	<0.01		
AFP (ng/mL)	1.00 (1.00, 1.00)	0.37			1.00 (1.00, 1.00)	0.60			1.00 (1.00, 1.00)	0.70		
Myosteatosis	0.96 (0.95, 0.98)	<0.01	0.97 (0.96, 1.00)	0.05	0.96 (0.95, 0.98)	<0.01	0.97 (0.95, 0.99)	<0.01	0.97 (0.95, 0.98)	<0.01	0.97 (0.95, 0.98)	<0.01
Sarcopenia	2.46 (1.71, 3.54)	<0.01	2.05 (1.41, 3.00)	<0.01	2.24 (1.66, 3.02)	<0.01	1.81 (1.29, 2.54)	<0.01	2.15 (1.67, 2.76)	<0.01	1.82 (1.30, 2.55)	<0.01
ARDS	1.55 (1.11, 2.16)	0.01	1.03 (0.72, 1.47)	0.89	1.41 (1.06, 1.88)	0.02	0.92 (0.67, 1.28)	0.63	1.58 (1.22, 2.02)	<0.01	0.94 (0.67, 1.30)	0.69
HE	1.97 (1.40, 2.78)	<0.01	1.56 (1.01, 2.25)	0.02	1.89 (1.40, 2.55)	0.00	1.57 (1.14, 2.20)	0.01	1.94 (1.48, 2.53)	<0.01	1.60 (1.16, 2.21)	0.01
Cirrhosis	0.78 (0.51, 1.20)	0.26			0.80 (0.56, 1.15)	0.23			0.75 (0.55, 1.03)	0.08		
Bleeding	1.49 (1.27, 2.52)	<0.00	1.00 (0.69, 1.44)	1.00	1.27 (0.94, 1.72)	0.11			1.74 (1.34, 2.25)	<0.01	0.87 (0.62, 1.21)	0.40
AKI	1.79 (1.27, 2.51)	0.01	1.17 (0.761.81)	0.47	1.64 (1.21, 2.23)	<0.01	1.15 (0.77, 1.72)	0.49	1.77 (1.35, 2.33)	<0.01	1.20 (0.85, 1.70)	0.30

Abbreviations: AFP, alpha‐fetoprotein; AKI, acute kidney injury; ARDS, acute respiratory distress syndrome; BMI, body mass index; CI, confidence interval; HE, hepatic encephalopathy; HR, hazard ratio; INR, international normalized ratio; WBC, white blood cell.

**Figure 4:**
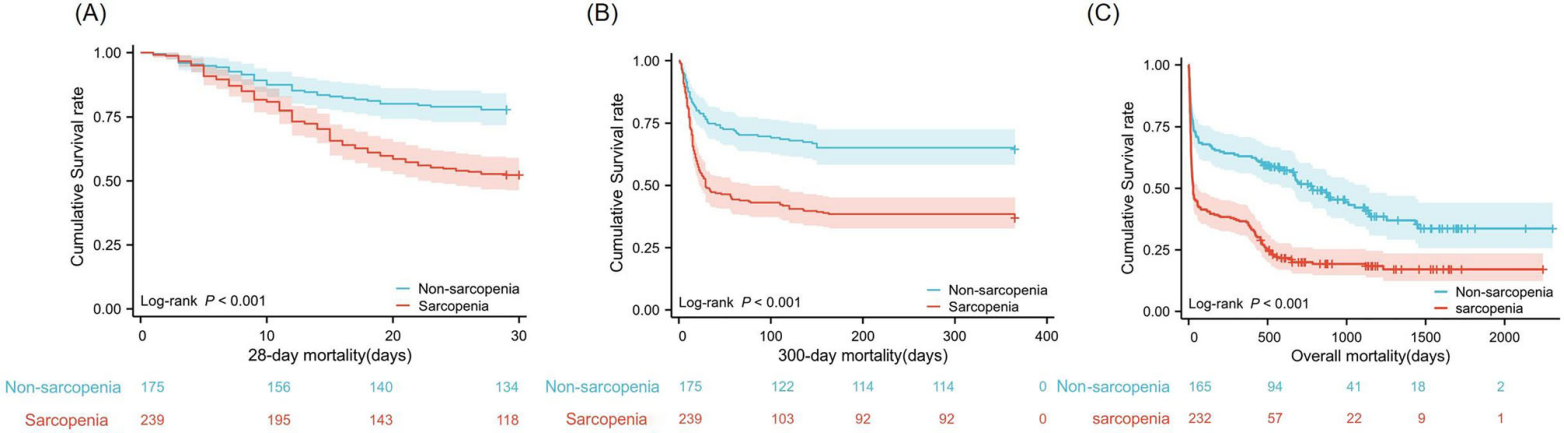
Kaplan–Meier curves of the survival rates of acute‐on‐chronic liver failure patients with or without sarcopenia for (A) 28‐day mortality, (B) 1‐year mortality and (C) overall mortality.

In the subsequent multivariate Cox regression analysis, sarcopenia, INR, muscle density and hepatic encephalopathy emerged as independent factors associated with mortality. Hazard ratios (HRs) for sarcopenia were 2.05 (1.41, 3.00) for 28‐day mortality, 1.81 (1.29, 2.54) for 1‐year mortality and 1.82 (1.30, 2.55) for overall mortality. The HRs for INR were 1.63 (1.34, 1.91) for 28‐day mortality, 1.62 (1.34, 1.97) for 1‐year mortality and 1.64 (1.36, 1.99) for overall mortality. The Kaplan–Meier curve further illustrated significantly higher 28‐day, 1‐year and overall mortality in the sarcopenia group than in the non‐sarcopenia group (*Figure* *4*).

## Discussion

Our retrospective analysis included 414 patients with ACLF that were admitted to our hospital over an 8‐year period. A comprehensive, long‐term follow‐up was conducted to evaluate their prognoses. Our findings reveal a significant association between sarcopenia and increased 28‐day, 1‐year and overall mortality rates in patients with ACLF. Specifically, patients with ACLF and sarcopenia had approximately 2.05‐, 1.81‐ and 1.82‐times higher risk of 28‐day, 1‐year and overall mortality, respectively, than those without sarcopenia. In both univariate and multivariate analyses, apart from sarcopenia, muscle density and INR were also found to correlate with the short‐ and long‐term prognoses of patients with ACLF. Notably, liver failure was identified as the primary cause of short‐term death in ACLF, whereas the proportion of deaths attributable to septic shock resulting from infection increased in the long term.

The Asian Working Group for Sarcopenia (AWGS) has proposed the use of the SMI, grip strength and usual gait speed for diagnosing sarcopenia.^21^ However, in the context of retrospective studies, assessing grip strength and gait speed in patients is often impractical. Consequently, previous studies have predominantly relied on the L3‐SMI to diagnose muscle wasting in patients with liver disease.^22^ The use of CT to measure SMI to assess sarcopenia has gained widespread acceptance.^23^, ^24^, ^25^ In the clinical setting, patients with ACLF frequently undergo CT because CT is used for both the evaluation and planning of interventions, such as liver transplantation. Thus, employing artificial intelligence (AI)‐based CT body composition analysis allows for the accurate determination of SMI. The threshold values of SMI for defining sarcopenia vary among different diseases. For example, the cut‐off value of SMI in sarcopenia for patients with sepsis was defined as L3‐SMI of <45.4 cm^2^/m^2^ for males and 34.4 cm^2^/m^2^ for females.^26^ Through literature review, we found that in most liver disease studies, cut‐off values of L3‐SMI for sarcopenia were defined as SMI of <50 cm^2^/m^2^ for males and <39 cm^2^/m^2^ for females.^7^, ^19^ Therefore, our study also used these thresholds to define sarcopenia.

Our study revealed that the sarcopenia group had a higher proportion of male individuals, which is consistent with prior research indicating a higher susceptibility of males to developing sarcopenia.^27^, ^28^ We observed that the sarcopenia group had higher CLIF‐SOFA scores than the non‐sarcopenia group, although no significant differences were found in the MELD and CTP scores. This observation suggests that the pathogenesis of ACLF may differ from that of patients with cirrhosis, with systemic inflammation potentially playing a pivotal role in the rapid onset of organ failure within a short time frame. Furthermore, traditional liver function scores such as MELD and CTP scores may not adequately reflect the severity of the condition, whereas organ function scores such as CLIF‐SOFA may provide a more accurate representation of the patient's clinical state.^29^, ^30^

Our investigation identified sarcopenia as an independent risk factor that was significantly associated with both short‐ and long‐term prognoses in patients with ACLF. Existing research on the prognostic value of sarcopenia in patients with ACLF has been largely limited to short‐term mortality, typically assessing 28‐ or 90‐day outcomes. Peng et al. recently reported the impact of sarcopenia on 90‐day mortality in a prospective cohort study.^31^ The study by Li et al. monitored 171 patients with ACLF for 90 days and found that sarcopenia did not decrease the survival rate of patients and did not investigate the long‐term impact of sarcopenia.^10^ Therefore, our study elucidated the impact of sarcopenia on long‐term overall mortality within a sizable population of patients with ACLF. We followed up and assessed 414 patients for more than 6 years, with a median follow‐up duration of 75.6 months among the surviving patients.

In patients with ACLF, portal systemic complications and impaired hepatic function often lead to reduced oral intake.^32^, ^33^ Systemic inflammation and hyperammonaemia can contribute to a heightened catabolic state and collectively lead to malnutrition and sarcopenia. Muscle mass depletion is a significant factor that suppresses amino acid and protein synthesis in response to biological changes in the immune system. On the other hand, in our research, 10 patients diagnosed with autoimmune hepatitis (AIH) underwent steroid treatment, and 9 of them developed sarcopenia. Patients with AIH often require steroid treatment.^34^ Prolonged steroid therapy may present an additional risk factor for sarcopenia due to the potential of glucocorticoids to induce oxidative stress in muscle tissue, thereby activating nuclear transcription factors in the forkhead box O family and exacerbating muscle atrophy.^35^ Currently, it is believed that a combination of resistance training exercise and a high‐protein diet can mitigate muscle atrophy, but a high‐protein diet raises the risk of hepatic encephalopathy.

Patients with sarcopenia are at an elevated risk of developing new infections and hepatic encephalopathy due to compromised immune function, further exacerbating the progression of ACLF.^36^, ^37^ Our research indicates that the primary cause of death in patients with ACLF is not solely the deterioration of liver function but also septic shock, which is a significant contributor to mortality in these patients, particularly 1 year after the onset of ACLF. Kumar et al.'s research discovered a notable increase in post‐operative sepsis among liver cirrhosis patients undergoing liver transplantation who had sarcopenia, potentially linked to protein and calorie deficiencies as well as compromised immunity.^38^ Although our study did not identify any differences in the distribution of mortality causes between sarcopenic and non‐sarcopenic groups, there was a growing proportion of deaths due to septic shock in the sarcopenia group over time. Furthermore, skeletal muscles play a critical role in overall energy and protein metabolism, and systemic inflammatory responses may trigger muscle breakdown, resulting in excessive energy consumption and eventual muscle loss, which in turn affects the immune system. Impaired immune response and a worse systemic immune status have been found in sarcopenia patients with cancer.^39^, ^40^ The question of whether sarcopenia leads to an increase in infection or if an increase in infection leads to sarcopenia requires further research in the future, as it could lead to the development of new treatment and prevention methods.

Recent studies have suggested that improvements in nutrition can reduce readmissions related to decompensation.^41^ Sinclair et al.'s study has shown that addressing sarcopenia can enhance the long‐term prognosis by increasing muscle mass in patients with liver cirrhosis.^42^ Therefore, sarcopenia treatment may be considered a crucial aspect in the management of patients with ACLF.

Our study has some limitations. First, it was a retrospective study. Although 414 patients were included and long‐term follow‐up was conducted, selection bias may still be present. In addition, we did not perform dynamic assessments of L3‐SMI levels, which leaves us uncertain about their clinical value in capturing dynamic changes over time. In the future, prospective studies will be utilized to actively track alterations in the SMI in an effort to elucidate these uncertain elements.

## Conclusions

In this study, the presence of sarcopenia was significantly associated with an increased mortality risk in both the short and long terms among patients diagnosed with ACLF. Therefore, prioritizing the ongoing monitoring and care of these patients is imperative, particularly regarding long‐term outcomes. Systematic monitoring of sarcopenia and interventions to enhance nutritional status may represent a promising avenue for the ongoing management of ACLF, aiming to improve treatment approaches and the overall patient prognosis.

## Conflict of interest statement

All authors declare that they have no conflict of interest.

## References

[1] Bajaj JS , O'Leary JG , Lai JC , Wong F , Long MD , Wong RJ , et al. Acute‐on‐chronic liver failure clinical guidelines. Am J Gastroenterol 2022;117:225–252. 35006099 10.14309/ajg.0000000000001595

[2] Durand F , Roux O , Weiss E , Francoz C . Acute‐on‐chronic liver failure: where do we stand? Liver Int 2021;41:128–136. 10.1111/liv.14855 34155793

[3] Cui Y‐L , Yan F , Wang Y‐B , Song X‐Q , Liu L , Lei X‐Z , et al. Nucleoside analogue can improve the long‐term prognosis of patients with hepatitis B virus infection‐associated acute on chronic liver failure. Dig Dis Sci 2010;55:2373–2380. 20512414 10.1007/s10620-010-1257-7

[4] Avolio AW , Nure E , Pompili M , Barbarino R , Basso M , Caccamo L , et al. Liver transplantation for hepatitis B virus patients: long‐term results of three therapeutic approaches. Transplant Proc 2008;40:1961–1964. 18675101 10.1016/j.transproceed.2008.05.071

[5] Cruz‐Jentoft AJ , Sayer AA . Sarcopenia. Lancet 2019;393:2636–2646. 31171417 10.1016/S0140-6736(19)31138-9

[6] Arroyo V , Moreau R , Jalan R . Acute‐on‐chronic liver failure. N Engl J Med 2020;382:2137–2145. 32459924 10.1056/NEJMra1914900

[7] Carey EJ , Lai JC , Sonnenday C , Tapper EB , Tandon P , Duarte‐Rojo A , et al. A North American expert opinion statement on sarcopenia in liver transplantation. Hepatology 2019;70:1816–1829. 31220351 10.1002/hep.30828 PMC6819202

[8] Kizilarslanoglu MC , Kuyumcu ME , Yesil Y , Halil M . Sarcopenia in critically ill patients. J Anesth 2016;30:884–890. 27376823 10.1007/s00540-016-2211-4

[9] Bai J , Xu M , Peng F , Gong J , Zhao J , Song X , et al. Skeletal muscle mass index as a predictor of long‐term cirrhosis onset in young non‐cirrhotic males with acute‐on‐chronic liver failure. Front Nutr 2022;9:1071373. 36618679 10.3389/fnut.2022.1071373 PMC9815435

[10] Li T , Xu M , Kong M , Song W , Duan Z , Chen Y . Use of skeletal muscle index as a predictor of short‐term mortality in patients with acute‐on‐chronic liver failure. Sci Rep 2021;11:12593. 34131260 10.1038/s41598-021-92087-1 PMC8206330

[11] Zheng M‐H , Shi K‐Q , Lin X‐F , Xiao D‐D , Chen L‐L , Liu W‐Y , et al. A model to predict 3‐month mortality risk of acute‐on‐chronic hepatitis B liver failure using artificial neural network. J Viral Hepat 2013;20:248–255. 23490369 10.1111/j.1365-2893.2012.01647.x

[12] Acute‐on‐chronic liver failure: consensus recommendations of the Asian Pacific Association for the Study of the Liver (APASL): an update—PubMed. https://pubmed.ncbi.nlm.nih.gov/31172417/. Accessed 8 June 2023.

[13] Malinchoc M , Kamath PS , Gordon FD , Peine CJ , Rank J , Ter Borg PCJ . A model to predict poor survival in patients undergoing transjugular intrahepatic portosystemic shunts. Hepatology 2000;31:864–871. 10733541 10.1053/he.2000.5852

[14] Liu P , Hsu C , Hsia C , Lee Y , Chiou Y , Huang Y , et al. ALBI and PALBI grade predict survival for HCC across treatment modalities and BCLC stages in the MELD Era. J Gastroenterol Hepatol 2017;32:879–886. 27696519 10.1111/jgh.13608

[15] Pugh RN , Murray‐Lyon IM , Dawson JL , Pietroni MC , Williams R . Transection of the oesophagus for bleeding oesophageal varices. Br J Surg 1973;60:646–649. 4541913 10.1002/bjs.1800600817

[16] Sy E , Ronco JJ , Searle R , Karvellas CJ . Prognostication of critically ill patients with acute‐on‐chronic liver failure using the Chronic Liver Failure‐Sequential Organ Failure Assessment: a Canadian retrospective study. J Crit Care 2016;36:234–239. 27569253 10.1016/j.jcrc.2016.08.003

[17] Shen Y , Luo L , Fu H , Xie L , Zhang W , Lu J , et al. Chest computed tomography‐derived muscle mass and quality indicators, in‐hospital outcomes, and costs in older inpatients. J Cachexia Sarcopenia Muscle 2022;13:966–975. 35178898 10.1002/jcsm.12948 PMC8977961

[18] Kim EH , Kim KW , Shin Y , Lee J , Ko Y , Kim Y‐J , et al. Reference data and T‐scores of lumbar skeletal muscle area and its skeletal muscle indices measured by CT scan in a healthy Korean population. J Gerontol A Biol Sci Med Sci 2021;76:265–271. 32179888 10.1093/gerona/glaa065

[19] Carey EJ , Lai JC , Wang CW , Dasarathy S , Lobach I , Montano‐Loza AJ , et al. A multicenter study to define sarcopenia in patients with end‐stage liver disease. Liver Transpl 2017;23:625–633. 28240805 10.1002/lt.24750 PMC5762612

[20] Nachit M , Horsmans Y , Summers RM , Leclercq IA , Pickhardt PJ . AI‐based CT body composition identifies myosteatosis as key mortality predictor in asymptomatic adults. Radiology 2023;307:e222008. 37191484 10.1148/radiol.222008 PMC10315523

[21] Correa‐de‐Araujo R , Hadley E . Skeletal muscle function deficit: a new terminology to embrace the evolving concepts of sarcopenia and age‐related muscle dysfunction. J Gerontol A Biol Sci Med Sci 2014;69:591–594. 24737562 10.1093/gerona/glt208 PMC3999854

[22] Fujiwara N , Nakagawa H , Kudo Y , Tateishi R , Taguri M , Watadani T , et al. Sarcopenia, intramuscular fat deposition, and visceral adiposity independently predict the outcomes of hepatocellular carcinoma. J Hepatol 2015;63:131–140. 25724366 10.1016/j.jhep.2015.02.031

[23] Montano‐Loza AJ , Duarte‐Rojo A , Meza‐Junco J , Baracos VE , Sawyer MB , Pang JXQ , et al. Inclusion of sarcopenia within MELD (MELD‐Sarcopenia) and the prediction of mortality in patients with cirrhosis. Clin Transl Gastroenterol 2015;6:e102. 26181291 10.1038/ctg.2015.31 PMC4816259

[24] Yao J , Zhou X , Yuan L , Niu LY , Zhang A , Shi H , et al. Prognostic value of the third lumbar skeletal muscle mass index in patients with liver cirrhosis and ascites. Clin Nutr 2020;39:1908–1913. 31472986 10.1016/j.clnu.2019.08.006

[25] Benmassaoud A , Roccarina D , Arico F , Leandro G , Yu B , Cheng F , et al. Sarcopenia does not worsen survival in patients with cirrhosis undergoing transjugular intrahepatic portosystemic shunt for refractory ascites. Am J Gastroenterol 2020;115:1911–1914. 33156111 10.14309/ajg.0000000000000959

[26] Oh HJ , Kim JH , Kim HR , Ahn JY , Jeong SJ , Ku NS , et al. The impact of sarcopenia on short‐term and long‐term mortality in patients with septic shock. J Cachexia Sarcopenia Muscle 2022;13:2054–2063. 35478354 10.1002/jcsm.12995 PMC9397556

[27] Tantai X , Liu Y , Yeo YH , Praktiknjo M , Mauro E , Hamaguchi Y , et al. Effect of sarcopenia on survival in patients with cirrhosis: a meta‐analysis. J Hepatol 2022;76:588–599. 34785325 10.1016/j.jhep.2021.11.006

[28] Prokopidis K , Affronti M , Testa GD , Ungar A , Cereda E , Smith L , et al. Sarcopenia increases mortality risk in liver transplantation: a systematic review and meta‐analysis. Panminerva Med 2023;66:47–54. 37539669 10.23736/S0031-0808.23.04863-2

[29] Hou Y , Zhang Q , Gao F , Mao D , Li J , Gong Z , et al. Artificial neural network‐based models used for predicting 28‐ and 90‐day mortality of patients with hepatitis B‐associated acute‐on‐chronic liver failure. BMC Gastroenterol 2020;20:75. 32188419 10.1186/s12876-020-01191-5 PMC7081680

[30] Katoonizadeh A , Decaestecker J , Wilmer A , Aerts R , Verslype C , Vansteenbergen W , et al. MELD score to predict outcome in adult patients with non‐acetaminophen‐induced acute liver failure. Liver Int 2007;27:329–334. 17355453 10.1111/j.1478-3231.2006.01429.x

[31] Peng H , Zhang Q , Luo L , Lei S , Xiong T , Long L , et al. A prognostic model of acute‐on‐chronic liver failure based on sarcopenia. Hepatol Int 2022;16:964–972. 35771410 10.1007/s12072-022-10363-2 PMC9349113

[32] Sinclair M , Gow PJ , Grossmann M , Angus PW . Review article: sarcopenia in cirrhosis—aetiology, implications and potential therapeutic interventions. Aliment Pharmacol Ther 2016;43:765–777. 26847265 10.1111/apt.13549

[33] Hayashi F , Matsumoto Y , Momoki C , Yuikawa M , Okada G , Hamakawa E , et al. Physical inactivity and insufficient dietary intake are associated with the frequency of sarcopenia in patients with compensated viral liver cirrhosis. Hepatol Res 2013;43:1264–1275. 23489325 10.1111/hepr.12085

[34] Granito A , Muratori P , Muratori L . Acute‐on‐chronic liver failure: a complex clinical entity in patients with autoimmune hepatitis. J Hepatol 2021;75:1503–1505. 34228991 10.1016/j.jhep.2021.06.035

[35] Klein GL . The effect of glucocorticoids on bone and muscle. Osteop Sarcop 2015;1:39–45. 10.1016/j.afos.2015.07.008 PMC4635469 26557727

[36] Wu J , Weisshaar N , Hotz‐Wagenblatt A , Madi A , Ma S , Mieg A , et al. Skeletal muscle antagonizes antiviral CD8^+^ T cell exhaustion. Sci Adv 2020;6:eaba3458. 32582853 10.1126/sciadv.aba3458 PMC7292629

[37] Ebadi M , Montano‐Loza AJ . Insights on clinical relevance of sarcopenia in patients with cirrhosis and sepsis. Liver Int 2018;38:786–788. 29702742 10.1111/liv.13720

[38] Kumar V , Benjamin J , Shasthry V , Subramanya Bharathy KG , Sinha PK , Kumar G , et al. Sarcopenia in cirrhosis: fallout on liver transplantation. J Clin Exp Hepatol 2020;10:467–476. 33029056 10.1016/j.jceh.2019.12.003 PMC7527849

[39] Wang P , Chen X , Liu Q , Yu Y , Xu L , Liu X , et al. Highlighting sarcopenia management for promoting surgical outcomes in esophageal cancers: evidence from a prospective cohort study. Int J Surg 2020;83:206–215. 33022414 10.1016/j.ijsu.2020.09.049

[40] Kitano Y , Yamashita Y , Saito Y , Nakagawa S , Okabe H , Imai K , et al. Sarcopenia affects systemic and local immune system and impacts postoperative outcome in patients with extrahepatic cholangiocarcinoma. World J Surg 2019;43:2271–2280. 31041559 10.1007/s00268-019-05013-y

[41] Reuter B , Shaw J , Hanson J , Tate V , Acharya C , Bajaj JS . Nutritional assessment in inpatients with cirrhosis can be improved after training and is associated with lower readmissions. Liver Transpl 2019;25:1790–1799. 31301208 10.1002/lt.25602 PMC7262968

[42] Sinclair M , Grossmann M , Hoermann R , Angus PW , Gow PJ . Testosterone therapy increases muscle mass in men with cirrhosis and low testosterone: a randomised controlled trial. J Hepatol 2016;65:906–913. 27312945 10.1016/j.jhep.2016.06.007

[43] von Haehling S , Morley JE , Coats AJS , Anker SD . Ethical guidelines for publishing in the Journal of Cachexia, Sarcopenia and Muscle: update 2021. J Cachexia Sarcopenia Muscle 2021;12:2259–2261. 34904399 10.1002/jcsm.12899 PMC8718061

